# Time taken to link newly identified HIV positive clients to care following a home-base index case HIV testing: Experience from two provinces in Zimbabwe

**DOI:** 10.1371/journal.pone.0201018

**Published:** 2018-08-22

**Authors:** Taurayi A. Tafuma, Nyikadzino Mahachi, Chengetai Dziwa, Peter Marowa, Tafara Moga, Tinashe Chimbidzikai, Auxillia Muchedzi, Tendai Nyagura, Mula Mpofu

**Affiliations:** 1 FHI 360, Harare, Zimbabwe; 2 USAID, Harare, Zimbabwe; 3 FHI 360, Pretoria, South Africa; The Ohio State University, UNITED STATES

## Abstract

**Background:**

Homebased index case HIV testing (HHTC) has shown higher uptake and good yield than traditional HIV testing methods. World Health Organization has called for increased operational research to evaluate HIV care processes particularly linkage to care. In this paper, we present project results of the time taken to link newly identified PLHIV to care after HHTC in the Manicaland and Midlands provinces of Zimbabwe.

**Methods:**

We retrospectively reviewed community-facility referral data from the Zimbabwe HIV Care and Treatment project for newly diagnosed PLHIV for the period March–September 2016. A referral slip was given to a client after receiving a positive HIV results and was presented and filed upon reaching a health facility. In July 2016, the project started working with trained expert clients to assist with linkage to care. Data was entered in a spreadsheet and then imported for descriptive statistical analysis with EpiInfo^TM^ Version 7.2.0.1. Odd ratios were used to identify factors associated with linkage to care within seven days.

**Results:**

Out of 1004 newly identified PLHIV between March and September 2016, 650 (64.7%) were linked to care. The median time taken to be linked to care was four days (Interquartile range 19 days). Overall, 63.1% (410) of PLHIV were linked to care within seven days of diagnosis and 85% within 30 days. PLHIV were more likely to be linked to care within seven days of diagnosis between July and September 2016 (OR = 4.1; p< 0.001), a period when ZHCT started working with expert clients to support linkage to care.

**Conclusion:**

HHTC resulted in almost 63% of newly diagnosed PLHIV being linked into care within seven days, and 85% within 30 days. Linkage to care within seven days was significantly associated with the period of engaging expert clients in the project. We recommend community based HIV testing programs to work with expert clients to ensure timely linkages of new PLHIV.

## Introduction

The extraordinary scale-up of Human Immunodeficiency Virus (HIV) testing, care and treatment programs in sub-Saharan Africa (SSA) has led to more people living with HIV (PLHIV) initiating antiretroviral therapy (ART) [1–3]. However, there have been some drawbacks on the overall effectiveness of HIV programs due to high levels of attrition across the HIV care continuum [3,4]. The success of an ART program depends on its ability to find, link, treat and maintain access to care for PLHIV [5]. Linkage to HIV care after HIV diagnosis is one of the most important steps in the HIV care continuum as it enables PLHIV to be assessed for their clinical status and be initiated on ART [1]. In SSA, it is reported that less than one-third of individuals testing HIV positive are linked to and engaged in care 12 months after diagnosis [3,6]. A district-wide homebased counseling and testing study in Uganda cited by van Rooyen [7] showed that only 11% of 11,359 PLHIV who were identified were initiated on ART. This low and untimely linkage to care results in further transmission of HIV in the communities. Linkage to and retention in care should be improved by strategies addressing individual, health care system, and societal barriers to HIV service utilization [5,8]

SSA’s countries have been reported to have daunting challenges to effectively track and monitor PLHIV as they move from one step to the other in the HIV continuum of care [2]. This has compromised the quality of HIV care programmes. Zimbabwe has been reported to have limited data on linkage to care, adherence, and viral suppression [4]. Access to HIV testing and counselling (HTC) services in Zimbabwe has mainly been through voluntary counselling and testing (VCT) approach or the provider initiated testing and counselling (PITC). Zimbabwe was recently funded to implement homebased index case testing and counselling (HHTC) through a mechanism called Zimbabwe HIV Care and Treatment (ZHCT) which is implemented by FHI360 and Plan International. Data from other countries on HHTC [9,10] has shown higher uptake of HTC and very good yield rate than traditional methods^10^ but there are associated challenges with linkage to care [5,6].

Recently, the World Health Organization has called for increased operational research to evaluate the HIV care process and further improve linkage to care, retention and adherence [11]. Current evidence suggests that HHTC is an economically sensible, acceptable and effective strategy for increasing HIV awareness [12,13] as it eliminates barriers associated with facility based testing. With improved linkage to care, this HHTC approach will be an ideal platform for the role out of “Test and Treat” strategy. Unfortunately, the reasons for suboptimal linkage are not well understood and require further attention if global and national targets are to be achieved [14] he few studies which have examined linkage to care, have defined linkage to care differently and evaluated it in a period of 1–12 months [3,6,7&^14^]. These timeframes need to be shortened so that they are in line with the “Test and Treat” approach which recommends rapid ART initiation (within seven days) [15]. The gap in literature is crucial to fill, because reviews on interventions to improve linkage and ART initiation are limited [14] yet a significant portion of HIV testing in the coming years will be community and home-based [6].

WHO recommendations suggest that the most likely points at which newly diagnosed PLHIV will become lost to care are between linkages from an HIV testing site to an HIV treatment site [16]. In this assessment, we defined linkage to care as confirmation of HIV diagnosis at community level and first HIV-specific health facility visit with confirmed registration in the pre-ART register for further clinical assessment and initiation on ART. In this paper, we present project results of the time taken to link newly identified PLHIV to care after HHTC in the Manicaland and Midlands provinces of Zimbabwe.

## Methods

### Setting and design

This study used data collected through routine ZHCT project implementation in Manicaland and Midlands provinces. FHI360 was awarded a five-year grant by USAID to implement at community level. The core activities include household index case testing, linkage to care, defaulter tracking and identification and formation of community ART refill groups as part of differentiated care model. The project started implementation in March 2016 in eight districts within these two provinces. The overall number of newly identified PLHIV was 2101 from 126 sites supported by the project between March-September 2016.

Certified nurses in the ZHCT project perform rapid HTC as per Zimbabwe National HTC guidelines as well as screening for TB and other non-communicable diseases. Clients diagnosed with HIV were referred to public health facilities using a referral slip for further management. The slip was filed at the referral facility and a carbon copy was left with the referring nurse. On a weekly basis, reconciliation was done to determine clients who would have completed referrals. Of note is that newly diagnosed clients could link to any facility of their choice in the country. When clients are linked into care for HIV services in Zimbabwe, they are given an ART number which is specific to that client and facility. The ART number assigned to a new client and date of registration was then recorded on the filed referral slip and this was also documented on the carbon copy of the referral slip as confirmation of completed referral.

All PLHIV in Zimbabwe are required to be registered in the Pre-ART register before they are initiated on ART in all public health facilities. Expert clients who were stable PLHIV already on ART and residing in the community within the supported health facilities were engaged after being recommended by the health facility management. By end of September 2016, the project had 188expert clients supporting the project as volunteers in escorting or reminding referred clients to complete their referrals. An expert patient training manual was developed and used to train the expert patients on the roles expected in the project. The topics covered during training includes project overview, skills on community and household entry, confidentiality, qualities of a good expert clients, use of project’s document tools and management of assets. The training was conducted over two days. Expert clients were capacitated with mobile phones with airtime and bicycles to improve communication and mobility within the community. These expert clients were fully engaged in July 2016 and were also assist facilities in tracking ART defaulters. At least one expert patient was assigned to a health facility and would work with the testing nurses as well as health facility staff. Newly diagnosed PLHIV were reminded to complete referrals through home visits or text messages. However, our reporting system was not tracking number of reminders which were conducted per client.

We retrospectively reviewed all referral tools thus the carbon copies of the referral slip with completed referrals. We extracted data-elements on newly diagnosed PLHIV who had been linked to care during March-September 2016 from 62 randomly selected health facilities supported by ZHCT. The supported health facilities in each district were arranged in an alphabetical order in excel and then random numbers generated were assigned to each site. Sites with random numbers were then arranged in an ascending order and the first half of the sites were selected for review.

### Data collection and analysis

All referral tools from the selected sites were reviewed and data-elements of interest were extracted. The referral tools had identifiable details of the clients and were only accessed by ZHCT Strategic Information (SI) officers for data extraction. These officers had routine access to other ZHCT project registers and were trained on good clinical practice and research ethics. All referral records of newly diagnosed PLHIV who completed linkage to care thus date tested and linked with a confirmed ART number within these sites were considered for review. Secondary data-elements of interest were age, sex, date of referral, suspicion of tuberculosis infection, province, and marital status. No identifiable data was collected from these project tools except that highlighted above. The local and FHI360 research committees waived the requirement of informed consent for this study. Data was entered in excel spreadsheet and further coded before it was then imported for descriptive statistical analysis with EpiInfo^TM^ Version 7.2.0.1. Multivariate logistic regression analysis was done to identify factors associated with linkage to care within seven days. Because we assessed the effect of a few independent variables, they were all included in the multivariate model. P values of 0.05 were considered statistically significant.

### Ethical review

This study was reviewed and approved by Medical and Research Council of Zimbabwe and the FHI360 institutional review board.

## Results

Out of 1004 newly identified PLHIV from these 62 health facilities, 650 (64.7%) were linked to care from March-September 2016. Their average age was 35years (Standard Deviation 12yrs). The median time to be linked to care after HIV diagnosis was four (4) days (Interquartile range 19 days). Majority of PLHIV linked to care were females (57.2%) and slightly above a third (36.5%) were reported to be married. A diagnosis of presumptive TB (suspect) was made in 37.5% of clients linked to care. Midlands province linked more PLHIV (57.2%) than Manicaland. Almost two thirds (67.7%) of PLHIV were linked to care between July and September 2016.

Overall, 63.1% (410) of PLHIV were linked to care within seven days of diagnosis and 80.0% (328) of these were linked between July and September 2016. Of the children aged below 18 years, 79.3% were linked within seven days of diagnosis while adults were 62.3% as shown (Table 1). Among males, 65.8% were linked to care within seven days while 61% were females.

**Table 1:** Summary of characteristics of clients linked to care.

		Number of days to be linked into HIV care (N = 650) n (%)					Total
		≤7 days	8–14 days	15-30days	31-60days	>60days	
**Age Category (years)**							
	**< 18 years**	23(79.3)	24(82.8)	27 (93.1)	27(93.1)	29(100)	**29**
	**18 + years**	387(62.3)	439 (70.7)	524 (84.4)	570 (91.8)	621 (100)	**621**
	**Total**	410 (63.1)	463 (71.2)	551(84.8)	597 (91.8)	650 (100)	**650**
**Sex**							
	**Male**	183 (65.8)	205 (73.7)	238 (85.5)	254 (91.3)	278 (100)	**278**
	**Female**	227 (61.0)	258 (69.3)	313 (84.2)	343 (92.2)	372 (100)	**372**
**Marital Status**							
	**Single-Adult**	86 (62.8)	100 (73)	111 (81.0)	124 (91)	137 (100)	**137**
	**Married**	141 (59.5)	165 (69.6)	194 (81.9)	215 (90.7)	237 (100)	**237**
	**Single-Child**	21 (75.0)	23 (82.1)	26 (92.8)	0 (0.0)	28 (100)	**28**
	**Not Recorded**	162 (65.3)	175 (70.6)	220 (88.7)	232 (93.5)	248 (100)	**248**
**Diagnosis of Presumptive Tuberculosis**							
	**No**	264 (65.0)	297 (73.1)	343 (84.5)	373 (91.9)	406 (100)	**406**
	**Yes**	146 (59.8)	166 (68.0)	208 (85.2)	224 (91.8)	244 (100)	**244**
**Province**							
	**Manicaland**	173 (62.2)	190 (68.3)	234 (84.2)	257 (92.4)	278 (100)	**278**
	**Midlands**	237 (63.7)	273 (73.4)	317 (85.2)	340 (91.4)	3372(100)	**372**

**Fig 1:**
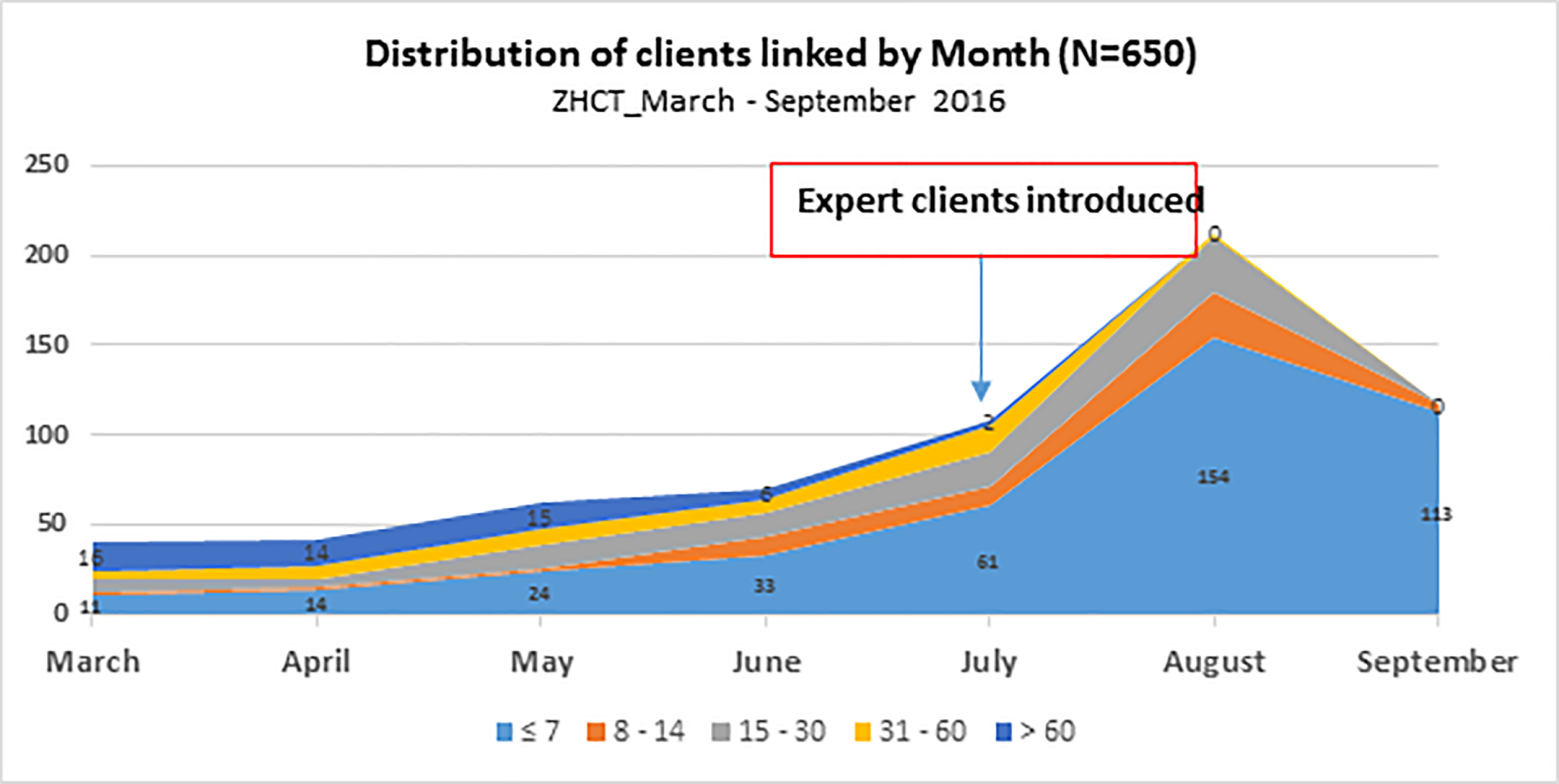
Distribution of clients linked within ZHCT by month and time it took to complete linkage.

Overall, 551 (84.8%) were linked into care within thirty days of HIV diagnosis and only 8.2% (53) were linked after 60 days as shown in Fig 1.

### Linkage duration by month

When HHTC was introduced through ZHCT in March 2016 less than 50 clients were linked, of which the majority took more than 30 days to have their linkage completed (Fig 1.). Over time, the number of clients being linked in the two provinces of Manicaland and Midlands continued to increase every month, while the time taken to complete linkage to care declined. Despite the continued upward trend in the number of clients linked, there was sharp increase in number linked during the month of July when expert clients were first utilized with the majority of the clients being linked within 7 days of diagnosis.

**Fig 2:**
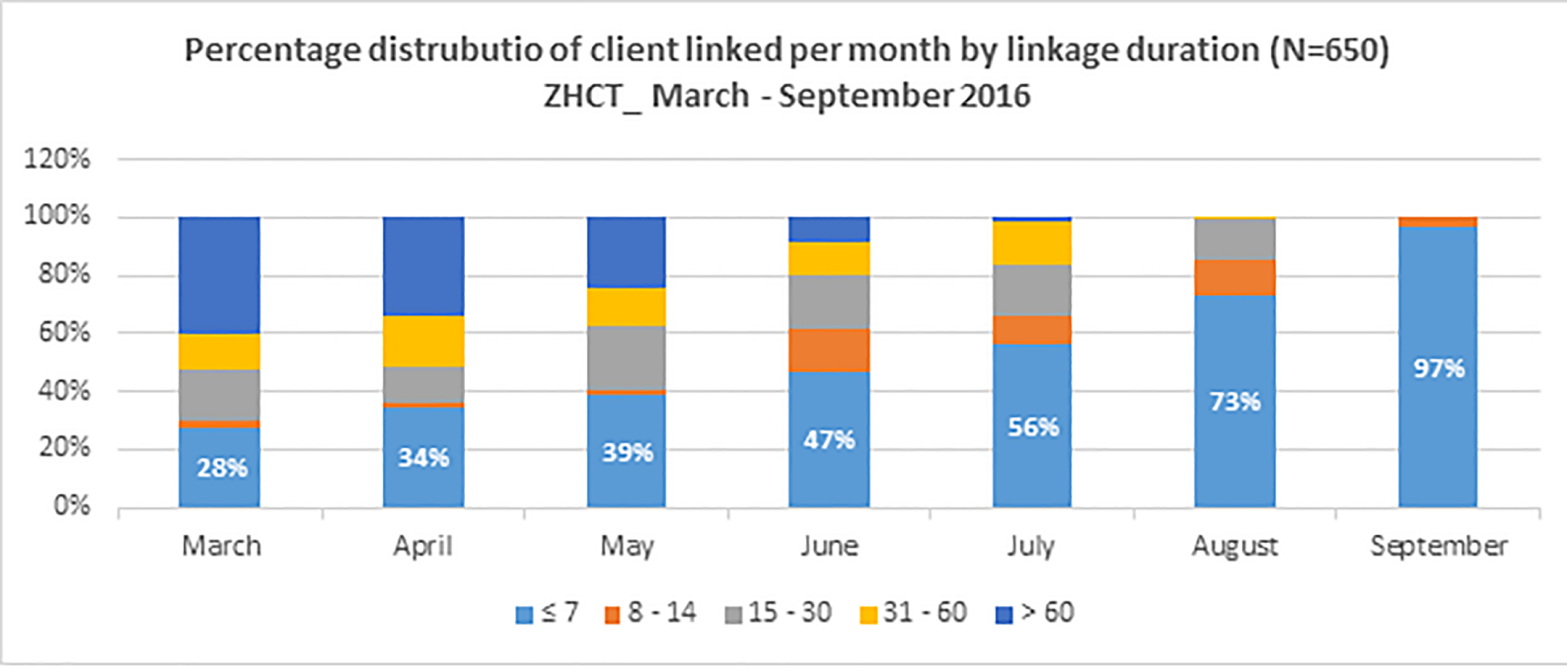
Percentage distribution of clients linked per month within ZHCT by time it took to complete linkage.

From *Fig 2*, HHTC reduced the linkage period over time, with only 28% of clients in the month of March having been linked within 7 days of diagnosis, and 60% taking more than 60 days to be linked. With time, linkage duration declined reaching 73% of clients within seven days after six months of implementation of HHTC during the month August, before reaching 97% during the month of September where all clients during that month were all linked within 14 days.

### Association between linkage and multiple factors

Newly identified PLHIV were 4.9 times more likely to be linked to care within seven days if they were diagnosed and referred at the period when expert clients were engaged (July-September 2016) (OR = 4.9; p< 0.001) and this is significant (*Table 2*).

**Table 2:** Multivariate associations with linkage to care within seven days.

Term	Odds Ratio	95%	C.I.
Age Logistic (<18/18+years)	2.32	0.93	5.77
Province (Midlands/Manicaland)	1.15	0.81	1.63
Period of referral (After July/Before July)	4.91	3.44	7.00
Sex (Male/Female)	1.29	0.91	1.83

From *Table 2*, there was also a positive association between duration of linkage (within seven days) and other factors such as age, sex and province, but this was not statistically significant.

## Discussion

Novel approaches such as HHTC have been considered ideal for identifying new PLHIV in communities [13]. However, the linkage rates have been low and the time taken to be linked to care has been documented in months [13] which will not be ideal for “Test and Treat” approach proposed by WHO [15]. Our findings demonstrate that HHTC can achieve very high levels of HIV care linkage and has evaluated the linkage time in days. When HHTC was introduced in March, linkage was less than 30% within 7 days and immediately increased to 41% within a similar period between April and June. After the engagement of expert clients, linkage of 75.1% was archived within 7 days which is very important under the current “Test and Treat” era. Early presentation at health facilities for clients diagnosed with HIV results in higher treatment response rates, lower mortality and reduces changes of HIV transmission at community level [7].

Community-based HIV screening strategies have been commended for reaching individuals who are likely not to access health care facilities due to lower perceived risk of HIV infection and their lack of physical symptoms [17,18,19]. Thus, such newly diagnosed PLHIV are likely to link to care at a time when their clinical status has deteriorated which could be several months from the time of diagnosis. The untimely linkage to care results in further transmission of HIV in communities. A study done in Mozambique indicated that only 57% of adults newly diagnosed with HIV were linked to care within 30 days [20] much lower than our findings (84.5%). Although, definitions of linkage to care vary from one study to the other [3], we still believe that our approach in this project results in higher linkage rates.

Early initiation of ART leads to improved clinical outcomes for PLHIV and have real-world impact on population health [19] Collaboration between community based HIV programs and health facilities should be strengthened so that PLHIV who are linked to care are quickly assessed and initiated on treatment. Zimbabwe has recently adopted the ‘Test and Treat’ approach and guidelines were released in December 2016 [21] and a country wide implementation was being proposed at the time of writing this paper. This ‘Test and Treat’ approach will result in timely initiation of ART for all PLHIV who are linked to care especially those who would be linked within seven days of diagnosis.

Linkage to care within seven days of diagnosis in this study which we consider as timely was not significantly associated with sex, marital status, age, presumptive diagnosis of TB and province. However, men as well as youth have been noted to link very late in care due to poor health seeking behavior and stigma respectively [19] Furthermore, low incidence to link to care among youth has been reported to be due to emotional and logistic challenges such as not accepting the new status, lack of social and financial support [13]. This then requires HIV programmers to develop tailored services for these subpopulations. The significant association (p<0.001) between linkage to care and the period July and September can be related to the ZHCT project starting to work with fully trained expert clients who assisted with linkage to care. Expert clients assisted with escorting PLHIV to health facilities or reminding them of the agreed appointments when the diagnosis was made. One qualitative study in Uganda highlighted inadequate counselling on “when and why” infected persons should make the link to care as a linkage barrier [22] In this project, expert clients work with ZHCT HIV testers on provision of psychosocial support. Lack of association with sex and age contrary to what has been noticed in other studies could be due to the support provided by these expert clients. The project recruited more males and younger expert clients to reach populations which had been having health seeking behavior challenges.

Although, we attribute improved linkage to care to the expert clients as has been noted in other studies [2,16], we further recommend prospective researches to validate this finding. Test and Treat approach was piloted in Manicaland in August and September, however, this seems not to have improved the proportions of clients linked within seven days of HIV diagnosis. Factors such as lack of understanding of HIV disease and associated care, forgetting appointments and the need for care while healthy have been noted to be addressed through reminders and enhanced counseling [3]. These could be the factors which the expert patients might have addressed when engaging newly diagnosed PLHIV resulting in them linking to care as early as within seven days. Also of note was that, a presumptive diagnosis of TB did not result in clients being more likely to be linked to care within seven days. This could be due to lack of knowledge among PLHIV that HIV/TB co-infection potentiate one another, accelerating the deterioration of immunological functions and resulting in premature death if untreated [23]. Health education on the effects of HIV/TB co-infection is required in these communities so that health seeking behavior is improved on those suspected of TB infection. If the ZHCT project can maintain this momentum of linking approximately 84.8% PLHIV within 30 days, it would perform within the expectation of well-resourced settings where linkage to care target within a month is set at 85% [24].

The strength of our study is that it reviewed data collected from a real program implementation other than a research controlled approach and this covered rural and urban settings with real geographic and other related public health system challenges. The project built on the approaches which Ministry of Health and Child Care of Zimbabwe has been implementing, that is engagement of community based volunteers as links between health facilities and communities. Our expert patients were capacitated the same way other volunteers were equipped (provision of airtime, bicycles and payment of stipends). With this, we feel our approach can be scaled up to other settings as long as targeted recruitment of volunteers is done so that existing gaps are addressed as we have done in this project.

There were some limitations to this assessment. Program data was used, which had some gaps in its completion and not specifically designed for research. This also limited ascertainment of client facilitators to linkage to care. However, our objectives were to elucidate the time taken to link to care and possible associated factors. Only few sites in the two provinces were considered which limits the generalizability of the findings however it highlights the importance of working with expert clients who provides peer support to newly diagnosed PLHIV. Also, this data might have been underreported as some clients are likely to access services outside the ZHCT catchment area.

## Conclusion

This is the first study to provide data on time taken for PLHIV to be linked into care in the two provinces of Zimbabwe from HHTC. HHTC resulted in almost 85% of newly diagnosed PLHIV linked into care within 30days and almost two thirds within seven days. Engaging expert clients to assist with linkage to care seems to improve the time taken for clients to be linked into care as was seen between July and September 2016. Considering that linkage to care is one of the important steps in the HIV continuum of care, we recommend HIV programs to work with PLHIV who are stable on ART so that they can provide peer support to newly diagnosed PLHIV.

## Supporting information

S1 File ZHCT linkaged referrals (003) Original Final Modified_submission.xls. (XLS)
